# Anchor-based evaluation of digital motor biomarkers from a 2-year observation in 100 patients with multiple sclerosis

**DOI:** 10.1007/s00415-025-13541-y

**Published:** 2025-12-04

**Authors:** Carla A. Ellinghaus, Hanna M. Röhling, Eva-Maria Dorsch, Pia S. Sperber, Radina Arsenova, Gilberto Solorza Buenrostro, Sophia Rekers, Tatiana Usnich, Patrick Schindler, Daniel Kroneberg, Judith Bellmann-Strobl, Frederike C. Oertel, Alexander U. Brandt, Martin Weygandt, Friedemann Paul, Sophie K. Piper, Tanja Schmitz-Hübsch

**Affiliations:** 1 Experimental and Clinical Research Center (ECRC), a Cooperation of Max-Delbrueck Center for Molecular Medicine and Charité – Universitätsmedizin Berlin, Clinical Neuroimmunology Group, Berlin, Germany; 2 Department of Psychosomatic Medicine, Charité – Universitätsmedizin Berlin, Berlin, Germany; 3 Department of Neurology and Experimental Neurology, Charité – Universitätsmedizin Berlin, Berlin, Germany; 4 Berlin School of Mind and Brain, Humboldt-Universität zu Berlin, Berlin, Germany; 5 Charité – Universitätsmedizin Berlin, NCRC – Neuroscience Clinical Research Center, Berlin, Germany; 6 Einstein Center Digital Future, Berlin, Germany; 7 Max-Delbrueck Center for Molecular Medicine, Berlin, Germany; 8 Charité – Universitätsmedizin Berlin, Institute of Biometry and Clinical Epidemiology, Berlin, Germany; 9 Charité – Universitätsmedizin Berlin, Institute of Medical Informatics, Berlin, Germany; 10 Charité – Universitätsmedizin Berlin, Clinical Trial Office (CTO), Berlin, Germany; 11 Motognosis GmbH, Berlin, Germany

**Keywords:** Multiple sclerosis, Progression, Minimal detectable change, Disease monitoring, Digital motor outcomes, Biomarkers

## Abstract

**Background:**

Description of disease progression in MS lack sensitive clinical outcomes. Digital motor outcomes (DMO) offer potential but require thorough validation.

**Objective:**

This 2-year observational study explores the sensitivity of 26 DMO derived from a task-based assessment protocol via RGB-D camera.

**Methods:**

One-hundred people with MS (pwMS) were enrolled, 96 completed follow-up. Expanded Disability Status Scale (EDSS) and 12-Item MS Walking Scale (MSWS-12) served as anchors for sensitivity by classifying progression, improvement or stable at 2 years, irrespective of intercurrent relapses. Primary analysis applied linear mixed modeling. Distribution-based thresholds (MDC) were calculated from stable subgroups.

**Results:**

Six DMO showed a significant increase over time in relation to stand-up/sit-movement (*β* = 0.01; *β* = 0.02), stepping asymmetry (*β* = 0.02; *β* = 0.02), trunk deflection while walking (*β* = 0.02) and trunk sway in closed-eyes stance (*β* = 0.02) (95% CI 0.001–0.038; all *p* < 0.05). EDSS (*n* = 22) and MSWS-12 (*n* = 11) progression groups were distinct. Time effects differed by anchor. For the EDSS progression group, the change in step width and sway velocity in closed-feet/open-eyes stance exceeded measurement error.

**Conclusions:**

Our findings yield a well-applicable set of DMO that may support definitions of disability accumulation in (early) pwMS. Establishing patient relevance remains challenging due to expectedly low clinical progression rates. The distinct patterns captured by EDSS and MSWS-12 definitions underline the need for multiple anchors when validating new biomarkers.

*Trial registration* ClinicalTrials.gov, NCT04993274, 2021-03-09 retrospectively registered.

**Supplementary Information:**

The online version contains supplementary material available at 10.1007/s00415-025-13541-y.

## Introduction

Monitoring of disease progression is a major challenge in multiple sclerosis (MS) as the accumulation of irreversible disability has emerged as a target of current therapeutic developments. Accordingly, accurate, sensitive and comprehensive disease severity measures are key to measure treatment effects [1]. In this context, motor function is of particular importance, as impairments have been linked to disability accrual [2]. Subtle changes in movement patterns have been identified early in the disease course as potential precursors of impaired mobility [3], and may occur associated with or independent of relapses (RAW/PIRA) [4]. However, such changes are not adequately captured in clinical ratings [5]. The Expanded Disability Status Scale (EDSS) is most commonly used to report disability progression, yet this established clinician-reported outcome has numerous limitations with respect to metric properties such as inter-rater reliability, linearity and sensitivity [6]. Quantitative performance markers could address the need for a more comprehensive and sensitive assessment. Instrumental assessments of motor function such as inertial sensors or markerless Red–Green–Blue-Depth (RGB-D) motion capture systems have proven to provide reliable measurements of gait and balance and have been proposed as potential early markers of disease progression [7–10]. Further, the COVID-19 pandemic has shown both the need and potential for digital motor outcomes (DMO) to enable remote monitoring of disease status in chronic diseases such as MS [11]. However, their broader application in research or clinical care is hindered by a lack of standardization and applicable benchmarks for interpretation [12]. Moreover, evidence regarding their responsiveness to worsening of motor function in MS remains limited. Few longitudinal studies reported changes in motor parameters in relation to established clinical markers [7, 10, 13], yet the predictive value and clinical relevance of DMO for disease monitoring remain insufficiently established.

To address this gap, we here provide results of a 2-year prospective observation in 100 people with MS (pwMS). This study used a previously described motion capture protocol based on RGB-D technology applied on a set of short motor tasks (Perceptive Assessment in Multiple Sclerosis, PASS-MS) [14], along with clinician-based ratings as well as patient report. Evidence from prior technical validation across different contexts included high accuracy and reliability for maximum gait speed [9, 14, 15], as well as reliability and validity for measures of postural control [16]. Moreover, we devised a standardized post hoc quality control process to enhance the reliability and validity of results [17] and a normative data set is available based on a mono-center observational study [18] as a reference point for patient cohorts.

This longitudinal study explores the sensitivity of DMO against anchors of clinical relevance. We hypothesized that a set of DMO, with maximum gait speed as the main outcome, reflects disease progression events captured by established EDSS criteria. To accommodate the patient’s perspective, analysis was paralleled using an alternative definition of progression events based on patient report of walking ability (MSWS-12) and published thresholds for clinically relevant change [19, 20]. The subgroups with stable EDSS and MSWS-12 served to derive first estimates of minimal detectable change for each motor outcome. Once a set of sensitive DMO is thus defined, further study may explore their specificity to specific MS symptoms.

## Methods

### Study design

This prospective observational study was conducted at an academic MS referral center (Charité—Universitätsmedizin Berlin) between 02/2019 and 11/2022. Study visits were scheduled 12 and 24 months apart, applying an identical assessment protocol. Where available, data from the same individuals from aligned observational studies using identical protocols were included to mitigate follow-up loss due to the COVID-19 pandemic (see Supplement and Supplementary Fig. S1). 100 pwMS were included based on: diagnosis of MS (RRMS, SPMS, and PPMS) according to 2017 McDonald criteria [21], ability to perform PASS-MS short walking tests unassisted or with unilateral assistance (EDDS < 6.5) and provision of written informed consent. Exclusion criteria were inability to follow test instructions for any reason and non-MS-related locomotor or balance dysfunction. Sample size was estimated to ensure a sufficient clinical progression rate [22].

### Study population

See Table 1.

**Table 1:** Description of study sample

Variables	*N*	Baseline	*N*	1YFU	*N*	2YFU	*N*	EDSS prog baseline	*N*	MSWS12 prog baseline
Age mean (SD)	100	41 (10)	63	44 (10)	96	43 (10)	22	41 (11)	11	43 (13)
Female (%*)*	100	39	63	38.1	96	37.5	22	77.3	11	27.3
Diagnosis (N)	100		63		96		22		11	
RRMS		80		47		76		12		9
SPMS		9		8		9		5		2
PPMS		3		3		3		1		0
CIS		8		5		8		4		0
EDSS median (range)	100	2 (0, 6.5)	63	2 (0, 6.5)	95	2 (0, 6.5)	22	1 (0, 6)	11	3 (1.5, 4.5)
0 (%)		14		11.1		12.6		40.9		/
1 (%)		15		7.9		12.6		13.6		/
1.5 (%)		19		22.2		21.1		9.1		27.3
2 (%)		13		12.7		14.7		9.1		18.2
2.5 (%)		8		9.5		5.3		4.5		/
3 (%)		11		7.9		9.5		4.5		9.1
3.5 (%)		8		9.5		8.4		4.5		27.3
4 (%)		4		4.8		6.3		4.5		9.1
4.5 (%)		1		1.6		/		/		9.1
5 (%)		1		1.6		1.1		/		/
5.5 (%)		1		3.2		2.1		/		/
6 (%)		4		3.2		3.2		9.1		/
6.5 (%)		1		4.8		3.2		/		/
SDMT mean (SD)	62	61.6 (10.3)	35	62.8 (11.6)	80	62.0 (13.1)	14	63.3 (13.6)	4	64.3 (12.6)
MSWS-12 rescale mean (SD)	78	16.7 (7.7)	49	17.8 (9.3)	89	18.1 (7.4)	14	25.7 (6.9)	11	14.8 (10.4)
HALEMS total mean (SD)	95	1.7 (0.5)	52	1.7 (0.6)	88	1.7 (0.5)	20	1.9 (0.4)	11	2.0 (0.4)
FSMC total score mean (SD)	95	50.2 (20.3)	52	52.1 (21.3)	87	50.3 (21.9)	20	53.3 (21.4)	11	57.4 (13.6)
Mild fatigue (%)		13.7		13.5		12.6		10		27.3
Moderate fatigue (%)		14.7		13.5		14.9		10		27.3
Severe fatigue (%)		30.5		36.5		33.3		45		36.4
BDI mean (SD)	94	5.7 (6.5)	52	4.8 (5.5)	89	5.5 (6.2)	19	5.5 (6.1)	11	5.8 (4.8)
BPI pain severity mean (SD)	76	0.9 (1.2)	27	0.9 (1.3)	61	1.1 (1.5)	16	0.6 (1.1)	8	1.3 (0.9)
Mild pain %		96.1		100		95.1		100		100
Moderate pain %		3.9		0		3.3		/		/
Severe pain %		0		0		1.7		/		/

*1YFU* 1-year follow-up, *2YFU* 2-year follow-up, *EDSS Prog* Assigned as EDSS progression, *MSWS12 Prog* Assigned as MSWS-12 progression, *SD* Standard deviation, *RRMS* Relapsing–remitting multiple sclerosis, *SPMS* Secondary progressive multiple sclerosis, *PPMS* Primary progressive multiple sclerosis, *CIS* Clinically isolated syndrome, *EDSS* Expanded Disability Status Scale, *SDMT* Symbol Digit Modalities Test, *MSWS-12 rescale* Twelve Item MS Walking Scale total score transformed to a scale from 0 to 100, *HALEMS total* Total score Hamburg Quality of Life Questionnaire in Multiple Sclerosis, *FSMC* Fatigue Scale for Motor and Cognitive Functions, *BDI* Beck Depression Inventory, *BPI* Brief Pain Inventor

### Clinical assessments

Neurological assessment included confirmation of diagnosis, EDSS ratings [23] and Symbol Digit Modalities Test (SDMT) [24]. Clinical ratings were performed by trained raters from the same study team.

### Performance outcomes

#### Digital outcomes of motor function

Trained operators instructed the PASS-MS protocol (Motognosis Labs, Version 1.2.0.0—1.4.0.2; Motognosis GmbH, Berlin, Germany) recorded with RGB-D sensor technology (Microsoft Kinect™ V2, version 2.0.14; Microsoft Corporation, Redmond, WA, USA) [18]. PASS-MS comprises ten tasks, of which the six used in this study are related to gait and balance functions (Table 2).

**Table 2:** Overview of included motor tasks in PASS-MS protocol

Task	Description
Postural control (POCO)	40 s closed stance with 20 s eyes open and 20 s eyes closed
Stepping in place (SIP)	40 s recording of stepping at a comfortable speed
Stand up and sit down (SAS)	Rising from a chair and sitting down with arms hanging loosely
Short line walk (SLW)	Walking about 5 m in tandem with a maximum of one misstep allowed
Short comfortable speed walk (SCSW)	Instructed as “your preferred speed, as if you would walk along a street”
Short maximum speed walk (SMSW)	Instructed as “as fast as possible while maintaining safety”

POCO and SIP were recorded once, while three recordings of SAS, SLW, SCSW and SMSW were obtained and averaged for analysis. Forty-two motor outcomes were generated based on previous work [9, 14–17].

#### Patient-reported outcomes (PRO)

The Twelve Item MS Walking Scale (MSWS-12) captures patients’ perception of walking ability [25, 26]. The sum score was transformed to a scale of 0–100, where higher scores indicate perceptions of more severe walking impairment [26]. Other PRO were applied for descriptive purposes only: Fatigue was assessed using the Fatigue Scale for Motor and Cognitive Functions (FSMC, 0 no fatigue to 100 most severe fatigue) [27], pain severity with the Brief Pain Inventory (BPI, 1–4 mild, 5–6 moderate, 7–10 severe) [28, 29], depressive symptoms with the Beck Depression Inventory II (BDI-II) [30] and MS-specific impairments in quality of life using the Hamburg Quality of Life Questionnaire in Multiple Sclerosis (HAQUAMS) [31].

### Statistical analysis

All statistical analyses were calculated using R Studio version 2023.03.0 + 386 [32]. The significance level was set at 0.05.

#### Anchor definitions of progression events

Disability progression at two years from baseline was defined using EDSS and MSWS-12 classifications and served as an anchor to evaluate DMO. For EDSS, progression events were defined according to commonly used conventions by a 1-step increase in EDSS for scores $\le$ 5.5 and a 0.5 step increase for scores > 5.5 [6]. Improvement was defined as respective decrease in EDSS [33] and stability as changes below these thresholds. Of note, the definition did not consider the occurrence of relapses and thus included RAW and PIRA.

Similarly, for MSWS-12, in the absence of established worsening criteria [20], we applied a threshold of 8 points change on the 0–100 transformed MSWS-12 scale, based on available evidence [19, 20]. Progression was defined as an increase of > 8 points, stability as changes between 0 and 8 points, and improvement as a decrease of > 8 points over 2 years.

#### Quality control and selection of DMO

The set of DMO was subject to post hoc quality control as described previously [17]. Recordings with relevant technical errors or deviations from the testing protocol were excluded. If a quality concern was identified for any test repetition, all recordings of that test were excluded for this participant. For SCSW and SMSW, the use of a unilateral walking aid had to be consistent across repetitions and study visits. Otherwise, all recordings of this test were excluded for this individual. For POCO, SIP and SLW, any assistance such as holding onto a wall or walking aid led to the exclusion of this visit’s recordings of that very task.

Intercorrelations were calculated for all motor outcomes to avoid redundancy. In case of a correlation of $\ge$ 0.8, one of the parameters was excluded from the analysis with preference for parameters with prior evidence of reliability or validity.

The remaining 26 motor outcomes were *z*-standardized using published normative data [18]. Where unavailable, normative data were calculated using the identical normative sample. The *z*-standardization allows direct comparison between all motor outcomes.

Descriptive analysis for baseline data used boxplots of *z*-scores to visualize the direction, extent and variability of difference in pwMS compared to the normative dataset. One-sample *z*-tests were calculated against a mean value of 0 (i.e. no deviation from the normative dataset).

#### Description of *z*-score changes for motor outcomes

Similarly, *z*-score changes over time were calculated as individual differences between 2-year and baseline *z*-score for each motor outcome. Resulting delta *z*-scores were summarized as group-level distributions and visualized as boxplots. A median *z*-score change of $\ge$ 0.5 was defined as threshold to indicate potentially relevant change [34].

#### Modelling of longitudinal change in motor outcomes

One linear Mixed Model (LMM) was calculated for each *z*-standardized motor outcome of the final dataset. This approach allows for adequately accounting for the dependence of the measurements due to the repeated measurement design and potential correlates of change. All available data points were included. Time as months since baseline was included as a fixed effect to assess the trajectory of motor outcomes over the observation period. Furthermore, fixed effects for progression status based on EDSS and MSWS-12, as well as their interactions with time were added. Observed progression events were dummy-coded, with 1 indicating progression and 0 indicating no progression. The interaction terms served to determine whether the rate of change in DMO differed depending on the respective anchor. Random intercepts for each subject were included to account for interindividual differences at baseline.

#### Estimates of minimal detectable change (MDC) for motor outcomes

The MDC was determined to quantify the smallest change in DMO that can reliably be distinguished from measurement error, serving as an important distribution-based metric of sensitivity to change [35]. MDC estimates were derived from the variance of changes in motor outcomes in subgroups classified as stable by EDSS or MSWS-12, respectively. Unstandardized values were used to retain the original measurement units, ensuring clinical relevance and practical interpretability of results.

MDC values were calculated based on the Standard Error of Measurement (SEM): MDC = 1.96 × √2 × SEM [36], where the SEM was derived from the residual variance and the Intraclass Correlation Coefficient (ICC): SEM = SD √(1-ICC) [37]. The ICC was calculated as the ratio of between-subject variance to the total variance as the sum of between-subject and within-subject variance across baseline and 2-year follow-up. To allow for a more precise estimation of variance, we again calculated LMM for each motor outcome based on the subgroups defined as stable over time [38] with a fixed effect for time and a random intercept to capture the between-subject variance, while the within-subject variance was estimated from the residual variance.

## Results

### Definitions of progression events

Data from 95 and 70 subjects were available to define progression for EDSS and MSWS-12, respectively. While the majority was consistently defined as stable, subgroups with progression events by either anchor criterion were distinct with overlap in only one subject (Table 3).

**Table 3:** Frequencies of assignment by definition of EDSS or MSWS-12, respectively

	EDSS improvement (*n* = 12)	EDSS stable (*n* = 61)	EDSS progression (*n* = 22)	NA
**MSWS-12 improvement** (*n* = **9**)	2	6	1	0
**MSWS-12 stabl**e (*n* = **50**)	7	33	9	1
**MSWS-12 progression** (*n* = **11**)	2	8	1	0
**NA**	1	14	11	

*EDSS* Expanded disability status scale, *MSWS-12* twelve item MS walking scale rescaled to 0–100

### Quality control and selection of motor outcomes

3396 recordings were inspected for data quality which led to the exclusion of 282 recordings (8.3%). Exclusions per test are provided in Supplementary Table S1.

Upon inspection of the correlation matrix (Supplementary Fig. S2), high correlations were found for outcomes of three tests: For POCO, ten parameters were excluded while parameters of angular velocity were retained [16]. For SAS, only parameters from the up condition i.e., sit-to-stand transition were considered due to higher technical robustness in QC. Due to the strong correlation between both hands in the up condition, a new variable was created as the mean of the right and left hand value. For SCSW, step length was excluded due to high intercorrelations as well as methodological considerations. This step resulted in 26 out of the initial 42 motor outcomes being used in the final analysis.

### Relation to normative data

**Fig. 1:**
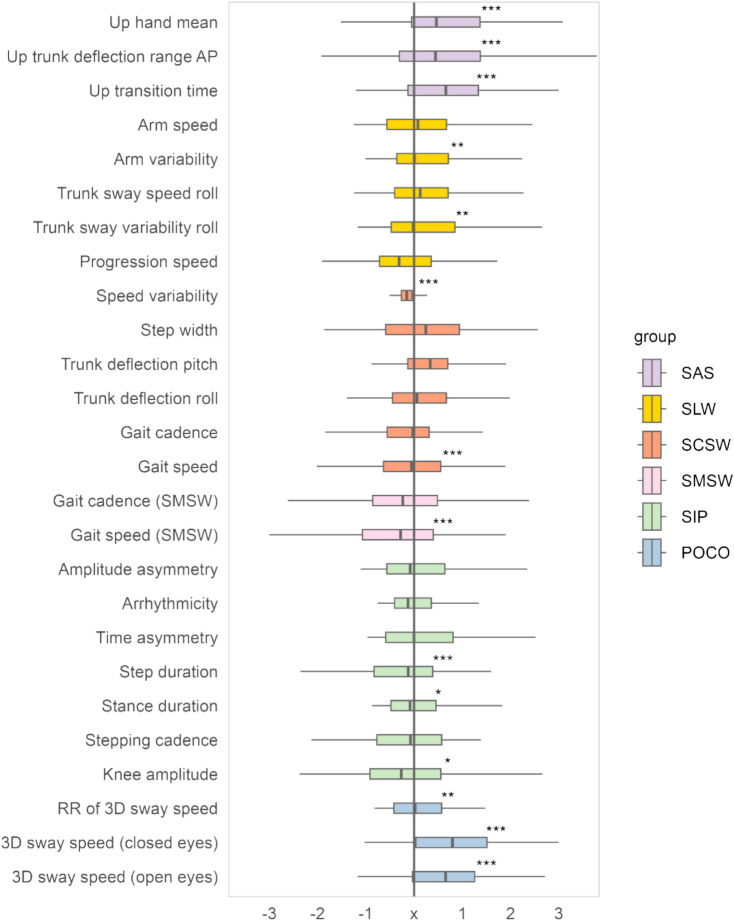
Boxplots of the final 26 *z*-score normalized motor outcomes at baseline (whole sample). Significant differences at group level from the mean value of the normative dataset are marked with asterisks. *SAS* Stand Up and Sit Down, *AP* anterior–posterior, *SLW* Short Line Walk, *SCSW* Short Comfortable Speed Walk, *SMSW* Short Maximum Speed Walk, *SIP* Stepping in Place, *POCO* Postural Control, *RR* Romberg Ratio,* x* mean value of the normative data

Fourteen out of 26 *z*-standardized motor outcomes differed from a normative dataset previously reported [18] (Fig. 1). This sample of predominantly mild disability (80% EDSS $\le$ 3) showed features of trunk instability (SAS, SLW, POCO) and increased compensatory arm movements (SLW, SAS), a markedly reduced gait speed for SMSW and SCSW and prolonged sit-to-stand transition time (SAS). Step and stance durations in SIP were increased, while knee amplitude of stepping was slightly reduced. Unexpectedly, speed variability (SCSW) was lower compared to the normative dataset, though mean difference was small. Moreover, no difference was seen for step width, trunk deflections (SCSW) or asymmetry measures for SIP.

### Description of *z*-score changes for motor outcomes

**Fig. 2:**
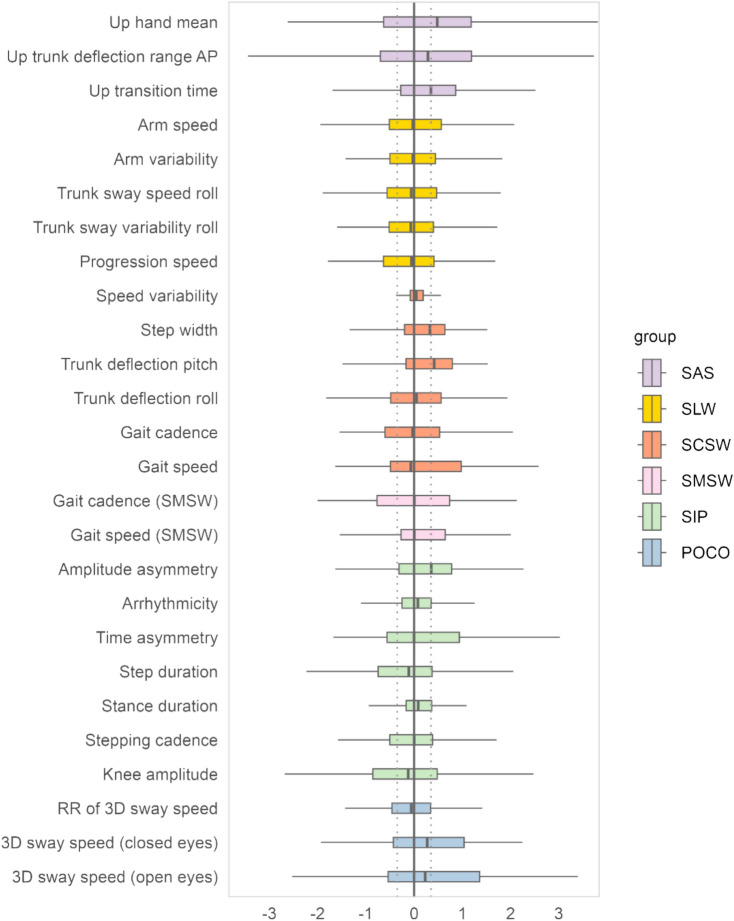
Boxplots of the *z*-score based change in motor outcomes at 2 years against baseline (whole group). The dotted lines indicate a standard deviation of 0.5 and − 0.5 as a reference for interpretation. *SAS* Stand Up and Sit Down, *AP* anterior posterior, *SLW* Short Line Walk, *SCSW* Short Comfortable Speed Walk, *SMSW* Short Maximum Speed Walk, *SIP* Stepping in Place, *POCO* Postural Control, *RR* Romberg Ratio

Changes in *z*-scores of the 26 motor outcomes between baseline and 2-year follow-up were overall small in magnitude (Fig. 2). However, there was high variance within-sample for some outcomes. Three parameters showed a potentially relevant change at group level > 0.5 SD (POCO postural sway velocity open and closed eyes conditions and SAS hand movement) and three more were just below the criterion (SAS up time, SCSW step width, SIP amplitude asymmetry). Of note, no substantial change in gait speed in either condition was seen at group level.

### Modelling of longitudinal change in motor outcomes

A total of 26 LMMs were calculated based on *z*-normalized DMO (Table 4). The model assumptions of homoscedasticity, linearity and normal distribution of residuals were sufficiently met across all analyzed parameters. For sway speed with closed eyes and the corresponding Romberg Ratio (POCO), as well as for time asymmetry (SIP), slight deviations from homoscedasticity were noted. Nevertheless, overall model fit was deemed acceptable for interpretation.

**Table 4:** Results of the *z*-score based linear mixed models

Parameter	Obsv.	Intercept (*β* (SE) [*t*, *p*])	Time (*β* (SE) [*t*, *p*])	EDSS prog (*β* (SE) [*t*, *p*])	Time:EDSS (*β* (SE) [*t*, *p*])	MSWS-12 prog (*β* (SE) [*t*, *p*])	Time:MSWS-12 (*β* (SE) [*t*, *p*])
**SAS**							
Up hand mean	229	0.39 (0.15) [2.52, 0.01*]	0.02 (0.01) [2.19, 0.03*]	0.04 (0.31) [0.11, 0.91]	0.01 (0.02) [0.57, 0.57]	0.33 (0.39) [0.83, 0.41]	− 0.01 (0.02) [− 0.56, 0.58]
Up trunk deflection range AP	229	0.36 (0.17) [2.14, 0.03*]	0.01 (0.01) [1.75, 0.08]	0.43 (0.34) [1.26, 0.21]	− 0.02 (0.02) [− 0.94, 0.35]	0.51 (0.44) [1.17, 0.24]	− 0.03 (0.02) [− 1.43, 0.15]
Up transition time	229	0.65 (0.2) [3.29, 0.00**]	0.01 (0.01) [2.09, 0.04*]	− 0.13 (0.39) [− 0.34, 0.74]	0.01 (0.01) [0.48, 0.63]	0.35 (0.51) [0.68, 0.49]	0.01 (0.02) [0.73, 0.46]
**SLW**							
Arm speed	207	0.78 (0.05) [1.45, 0.15]	0.00 (0.00) [0.75, 0.46]	− 0.09 (0.10) [− 0.95, 0.34]	0.00 (0.01) [0.03, 0.98]	0.11 (0.13) [0.81, 0.42]	0.00 (0.01) [0.05, 0.96]
Arm variability	207	0.20 (0.01) [1.68, 0.09]	0.00 (0.00) [0.16, 0.87]	− 0.02 (0.06) [− 0.4, 0.69]	0.00 (0.00) [0.43, 0.67]	0.16 (0.07) [2.15, 0.03*]	0.00 (0.00) [0.4, 0.69]
Trunk sway speed roll	207	0.41 (0.15) [2.79, 0.01**]	− 0.02 (0.01) [− 0.14, 0.89]	− 0.06 (0.29) [− 0.21, 0.84]	0.00 (0.01) [0.24, 0.81]	0.44 (0.38) [1.17, 0.24]	− 0.01 (0.02) [− 0.22, 0.83]
Trunk sway variability roll	207	0.47 (0.17) [2.75, 0.01**]	− 0.01 (0.01) [− 0.89, 0.37]	0.15 (0.35) [0.42, 0.68]	0.02 (0.01) [1.31, 0.19]	0.62 (0.44) [1.40, 0.16]	0.00 (0.02) [0.05, 0.96]
Progression speed	207	0.18 (0.14) [1.22, 0.23]	− 0.05 (0.01) [− 0.47, 0.64]	− 0.53 (0.29) [− 1.8, 0.07]	0.00 (0.01) [− 0.17, 0.87]	− 0.75 (0.37) [− 2.02, 0.05*]	0.02 (0.02) [1.32, 0.19]
**SCSW**							
Speed variability	209	− 0.16 (0.03) [− 6.16, 0.00**]	0.00 (0.00) [1.52, 0.13]	− 0.1 (0.05) [− 2.09, 0.04*]	0.00 (0.00) [1.01, 0.32]	− 0.01 (0.06) [− 0.09, 0.92]	− 0.00 (0.00) [− 0.66, 0.51]
Step width	207	− 0.02 (0.15) [− 0.12, 0.91]	0.01 (0.01) [0.83, 0.41]	0.25 (0.29) [0.86, 0.39]	0.03 (0.01) [2.14, 0.04*]	0.04 (0.37) [0.11, 0.91]	0.00 (0.02) [0.22, 0.82]
Trunk deflection pitch	209	0.07 (0.11) [0.64, 0.52]	0.02 (0.01) [3.41, 0.00***]	0.24 (0.21) [1.13, 0.25]	− 0.01 (0.01) [− 1.25, 0.21]	0.3 (0.26) [1.16, 0.25]	− 0.03 (0.01) [− 1.79, 0.08]
Trunk deflection roll	209	0.17 (0.16) [1.03, 0.3]	0.01 (0.01) [0.74, 0.46]	0.45 (0.31) [1.45, 0.15]	− 0.02 (0.01) [− 1.28, 0.2]	− 0.05 (0.39) [− 0.13, 0.89]	0.01 (0.02) [0.33, 0.75]
Gait cadence	208	− 0.25 (0.16) [− 1.5, 0.14]	0.01 (0.01) [0.99, 0.33]	− 0.28 (0.32) [− 0.89, 0.37]	− 0.01 (0.01) [− 0.48, 0.63]	− 0.06 (0.41) [− 0.15, 0.88]	0.00 (0.02) [0.07, 0.95]
Gait speed	209	− 0.29 (0.16) [− 1.89, 0.06]	0.01 (0.01) [1.51, 0.14]	− 0.58 (0.31) [− 1.89, 0.06]	− 0.00 (0.01) [− 0.2, 0.84]	0.05 (0.39) [0.13, 0.9]	− 0.01 (0.02) [− 0.84, 0.4]
**SMSW**							
Gait cadence	214	− 0.16 (0.17) [− 0.96, 0.34]	0.01 (0.01) [1.32, 0.19]	− 0.46 (0.33) [− 1.39, 0.17]	− 0.02 (0.01) [− 1.31, 0.19]	0.17 (0.44) [0.38, 0.7]	− 0.01 (0.02) [− 0.82, 0.41]
Gait speed	224	− 0.54 (0.19) [− 2.87, 0.02*]	0.00 (0.01) [0.62, 0.54]	− 0.86 (0.38) [− 2.28, 0.02*]	0.01 (0.01) [1.19, 0.24]	− 0.03 (0.49) [− 0.06, 0.95]	0.00 (0.01) [0.08, 0.94]
**SIP**							
Amplitude asymmetry	209	0.27 (0.26) [1.06, 0.29]	0.02 (0.01) [2.35, 0.02*]	0.03 (0.49) [0.05, 0.96]	− 0.00 (0.02) [− 0.21, 0.84]	0.12 (0.67) [0.18, 0.86]	− 0.02 (0.02) [− 0.79, 0.43]
Arrhythmicity	209	0.03 (0.61) [0.28, 0.78]	0.03 (0.03) [0.97, 0.33]	1.7 (1.17) [1.46, 0.15]	− 0.02 (0.05) [− 0.4, 0.69]	− 0.19 (1.58) [− 0.12, 0.91]	0.02 (0.08) [0.31, 0.76]
Time asymmetry	209	0.53 (0.29) [1.8, 0.08]	0.02 (0.01) [2.05, 0.04*]	− 0.01 (0.56) [0.00, 0.99]	− 0.03 (0.02) [− 2.04, 0.04*]	0.03 (0.75) [0.04, 0.97]	0.01 (0.02) [0.61, 0.54]
Step duration	209	0.11 (0.16) [0.73, 0.47]	− 0.01 (0.01) [− 1.03, 0.3]	0.09 (0.29) [0.31, 0.76]	− 0.01 (0.01) [− 0.69, 0.49]	− 0.33 (0.41) [− 0.83, 0.41]	0.02 (0.02) [1.07, 0.29]
Stance duration	209	0.27 (0.24) [1.09, 0.28]	0.01 (0.01) [0.98, 0.33]	− 0.01 (0.47) [− 0.02, 0.98]	− 0.01 (0.02) [− 0.44, 0.66]	− 0.13 (0.64) [− 0.2, 0.84]	0.00 (0.03) [0.08, 0.94]
Stepping cadence	209	− 0.17 (0.15) [− 1.13, 0.26]	0.00 (0.01) [0.01, 0.99]	0.01 (0.29) [0.05, 0.96]	0.00 (0.01) [0.26, 0.79]	0.16 (0.39) [0.41, 0.68]	− 0.01 (0.02) [− 0.77, 0.44]
Knee amplitude	209	0.04 (0.15) [0.27, 0.79]	− 0.01 (0.01) [− 1.17, 0.25]	− 0.02 (0.29) [− 0.06, 0.96]	− 0.01 (0.01) [− 0.69, 0.49]	− 0.49 (0.39) [− 1.27, 0.21]	0.00 (0.02) [0.19, 0.84]
**POCO**							
RR of 3D sway speed	212	0.23 (0.16) [1.44, 0.15]	0.01 (0.01) [0.85, 0.39]	0.15 (0.33) [0.46, 0.65]	− 0.02 (0.02) [− 1.17, 0.24]	0.72 (0.27) [2.65, 0.01**]	− 0.05 (0.02) [− 2.31, 0.02*]
3D sway speed (ce)	212	0.49 (0.27) [2.71, 0.01**]	0.02 (0.01) [2.83, 0.01**]	0.03 (0.54) [0.06, 0.95]	0.01 (0.02) [0.69, 0.49]	1.41 (0.46) [3.07, 0.00**]	− 0.08 (0.03) [− 2.89, 0.01**]
3D sway speed (oe)	212	0.53 (0.21) [2.48, 0.01*]	0.02 (0.01) [1.71, 0.09]	− 0.33 (0.43) [− 0.77, 0.44]	0.05 (0.02) [2.26, 0.03*]	0.67 (0.54) [1.24, 0.22]	− 0.02 (0.03) [− 0.85, 0.39]

*Obsv* number of observations (total count of recordings for analysis after QC processing), *β* fixed effects estimate, *SE* standard error, *t t*-value, *p p*-value, *Time* months since baseline, *EDSS Prog* assigned as EDSS progression, *MSWS12 Prog* assigned as MSWS-12 progression, *Time·EDSS* interaction between time and EDSS progression event, *Time·MSWS-12* interaction between time and MSWS-12 progression event, *SAS* Stand Up and Sit Down, *SLW* Short Line Walk, *SCSW* Short Comfortable Speed Walk, *SMSW* Short Maximum Speed Walk, *SIP* Stepping in Place, *POCO* Postural Control, *RR* Romberg Ratio, *ce* closed eyes, *oe* open eyes

*(*p* < 0.05), **(*p* < 0.001), ***(*p* < 0.0001)

#### Time

**Fig. 3:**
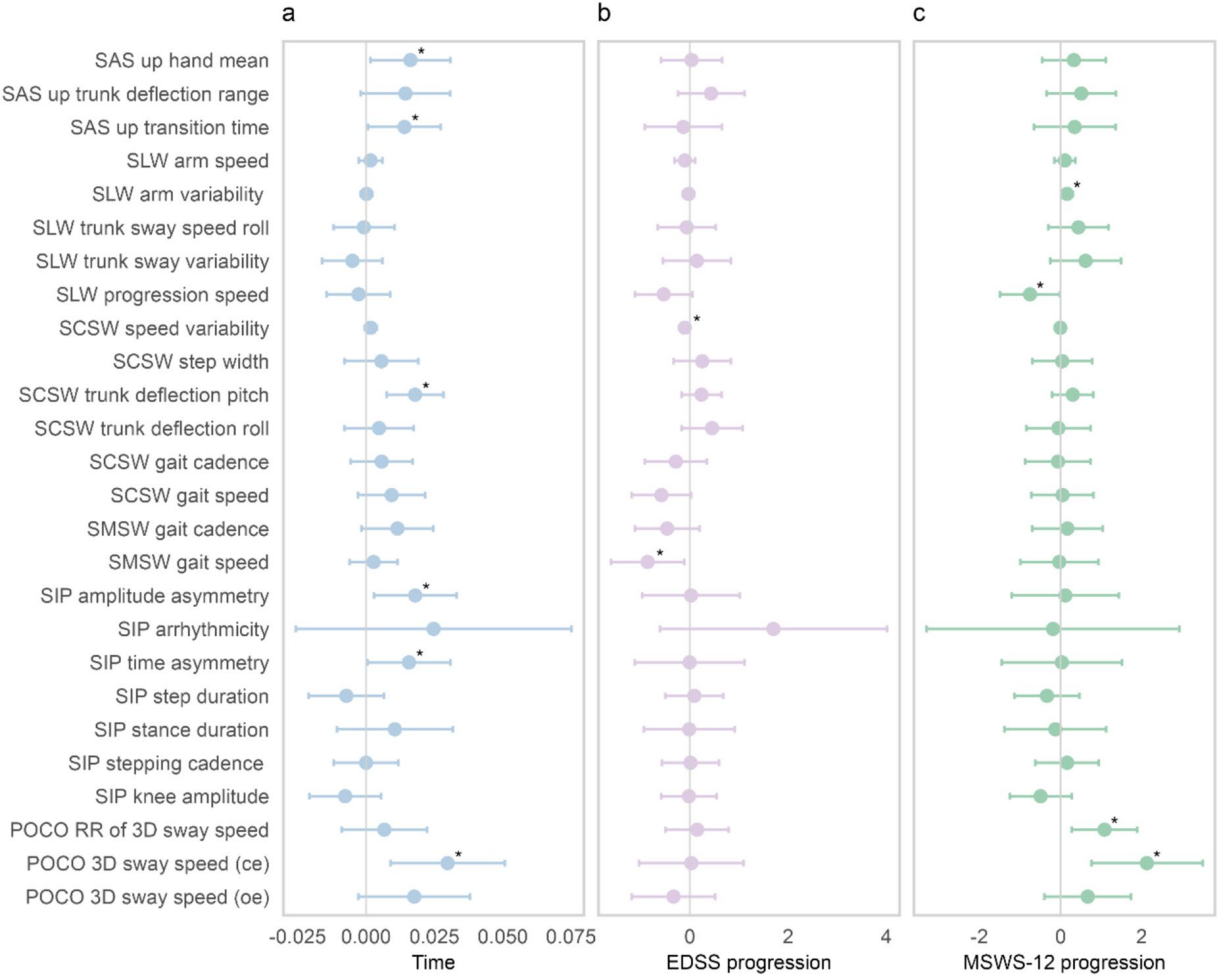
Overview of the estimated main effect sizes across all 26 motor outcomes included in the LMM. The plots display the estimated standardized effect sizes, expressed in standard deviation units, for **A** time, **B** the impact of an EDSS progression event, and **C** an MSWS-12 progression event. Dots represent the estimated effect sizes, and horizontal lines indicate the 95% confidence intervals. Significant effects are highlighted by asterisks. **A** The *x*-axis shows the estimated change per month since baseline in standard deviation units. **B** The *x*-axis shows the standardized group difference between patients with and without EDSS progression. **C** Similarly, the *x*-axis shows the standardized difference between patients with and without MSWS-12 progression

Significant time effects emerged for six motor outcomes across the whole group (Fig. 3a). For SAS, hand/arm movement and up transition time increased by 0.38 SD and 0.34 SD over 24 months, respectively. Similar increases were observed for trunk deflection pitch (SCSW) (0.43 SD), SIP amplitude asymmetry (0.43 SD) and time asymmetry (0.38 SD). 3D sway speed with eyes closed (POCO) increased by 0.46 SD over the same period.

#### EDSS progression event

Effects of EDSS progression events were estimated as lower speed variability (SCSW) and reduced maximum gait speed (SMSW) (Fig. 3b). Trends were seen towards a lower comfortable gait speed (SCSW) and progression speed (SLW).

#### MSWS-12 progression event

Effects of the alternative anchor criterion were seen on four motor outcomes (Fig. 3c): those with a progression event displayed higher arm variability and a lower progression speed in SLW and increases in 3D sway speed with closed eyes and the Romberg Ratio of 3D sway speed.

#### Interactions EDSS progression and time

Three motor outcomes showed differential change over time depending on EDSS progression status. Subjects with progression demonstrated an increase in step width (SCSW) of 0.84 SD over 24 months compared to 0.14 SD in those without. Similarly, 3D sway speed (oe) (POCO) increased by 1.5 SD over 24 months in the progressions group versus 0.42 SD in those without. For time asymmetry (SIP), subjects with progression unexpectedly showed a decline of − 0.36 SD over 24 months, while those without increased by 0.38 SD.

#### Interactions MSWS-12 progression and time

Effects were seen for two motor outcomes of POCO: For 3D sway speed (ce), subjects with MSWS-12 progression unexpectedly showed a decline of 1.13 SD over two years, while those without increased by 0.7 SD. A similar pattern emerged for the RR of sway speed, showing a decrease of 0.94 SD in the progression group and a slight increase of 0.14 SD in the non-progressing group over two years. Comparable trends were observed for trunk deflection pitch (SCSW).

### MDC

The MDC was calculated twice for each motor outcome (Table 5). First, from 61 subjects classified as stable by EDSS and, second, from 50 subjects classified as stable according to MSWS-12. Resulting MDC values were quite consistent between both derivatives, except for step width (SCSW) and gait cadence (SMSW) with higher MDC derived from those with stable MSWS-12. In the subset with a progression event defined by EDSS, mean change in 3D sway speed oe (POCO) and step width (SCSW) exceeded the MDC. For subjects with progression events defined by MSWS-12, none of the observed changes in motor outcomes exceeded the MDC.

**Table 5:** Minimal detectable change based on subgroups defined as stable on EDSS or MSWS-12, respectively

Motor outcomes	Baseline total mean (SD)	EDSS				MSWS-12			
		ICC	SEM	MDC	Δ prog (*p*)	ICC	SEM	MDC	Δ prog (*p*)
**SAS**									
Up hand mean (m)	0.35 (0.13)	0.49	0.07	0.19	0.07 (0.28)	0.49	0.07	0.19	0.00 (0.95)
Up trunk deflection range (m)	0.4 (0.1)	0.29	0.07	0.19	0 (0.91)	0.38	0.06	0.17	− 0.04 (0.23)
Up transition time (s)	1.64 (0.29)	0.66	0.11	0.29	0.1 (0.07)	0.73	0.08	0.22	0.09 (0.98)
**SLW**									
Arm speed (°/s)	10.25 (2.72)	0.49	1.55	4.29	0.32 (0.72)	0.53	1.42	3.92	− 0.32 (0.81)
Arm variability (°)	1.69 (0.74)	0.67	0.26	0.71	0.09 (0.59)	0.59	0.25	0.69	0.07 (0.78)
Trunk sway speed roll (°/s)	6.39 (2.08)	0.59	0.89	2.49	0.08 (0.91)	0.62	0.81	2.25	− 0.68 (0.29)
Trunk sway variability roll (°)	2.2 (1.03)	0.77	0.25	0.68	0.22 (0.27)	0.72	0.27	0.74	− 0.16 (0.41)
Progression speed (m/s)	0.35 (0.11)	0.58	0.05	0.14	− 0.02 (0.35)	0.61	0.04	0.12	0.02 (0.66)
**SCSW**									
Speed variability (m/frame)	0.01 (0)	0.57	0.00	0.00	0.00 (0.22)	0.56	0.00	0.00	0.00 (0.91)
Step width (cm)	10.45 (2.85)	0.69	0.91	2.53	2.94 (0.03*)	0.36	2.34	6.48	1.06 (0.25)
Trunk deflection pitch (°)	6.26 (2.52)	0.43	1.34	3.71	0.48 (0.38)	0.45	1.21	3.35	− 0.04 (0.11)
Trunk deflection roll (°)	3.55 (1.83)	0.59	0.73	2.02	− 0.22 (0.46)	0.48	0.78	2.17	0.31 (0.08)
Gait cadence (steps/min)	108.41 (13.8)	0.67	4.25	11.78	− 2.06 (0.37)	0.64	4.35	12.06	1.54 (0.75)
Gait speed (m/s)	1.09 (0.22)	0.59	0.09	0.234	0.01 (0.89)	0.62	0.08	0.22	− 0.02 (0.79)
**SMSW**									
Gait cadence (steps/min)	139.7 (18.54)	0.64	8.07	12.37	− 3.13 (0.59)	0.51	10.59	29.36	− 4.51 (0.41)
Gait speed (m/s)	1.55 (0.26)	0.83	0.04	0.12	0.07 (0.08)	0.82	0.05	0.14	0.01 (0.79)
**SIP**									
Amplitude asymmetry (%)	11.3 (14.55)	0.81	3.73	10.34	3.13 (0.39)	0.73	4.21	11.67	4.34 (0.16)
Arrhythmicity (%)	8.13 (3.9)	0.41	2.09	5.79	0.23 (0.98)	0.59	1.55	4.29	1.00 (0.46)
Time asymmetry (%)	6.48 (8.27)	0.89	1.33	3.68	− 1.7 (0.23)	0.71	2.61	7.24	3.46 (0.44)
Step duration (s)	0.84 (0.12)	0.59	0.06	0.15	− 0.03 (0.36)	0.53	0.06	0.17	0.01 (0.85)
Stance duration (s)	0.43 (0.21)	0.51	0.16	0.44	0.00 (0.86)	0.72	0.06	0.17	0.01 (0.72)
Stepping cadence (steps/min)	95.16 (19.32)	0.72	5.59	15.49	0.39 (0.94)	0.64	6.98	19.34	− 3.64 (0.62)
Knee amplitude (m)	0.18 (0.07)	0.5	0.03	0.09	− 0.03 (0.25)	0.53	0.03	0.09	− 0.02 (0.68)
**POCO**									
RR of 3D sway speed (n.u.)	0.69 (0.24)	0.46	0.38	1.06	− 0.16 (0.23)	0.18	0.46	1.27	− 0.45 (0.25)
3D sway speed (ce) (°/s)	0.41 (0.17)	0.71	0.07	0.18	0.12 (0.03*)	0.63	0.07	0.19	− 0.11 (0.09)
3D sway speed (oe) (°/s)	0.025 (0.11)	0.61	0.04	0.12	0.12 (0.02*)	0.42	0.07	0.2	− 0.03 (0.75)

MDC and mean changes are reported in original units of measurement

*SD* standard deviation, *ICC* intraclass correlation of stable EDSS/MSWS-12 group, *SEM* standard error of measurement of stable EDSS/MSWS-12 group, *MDC* minimal detectable change, *Δ Prog* mean change in EDSS/MSWS-12 progression group between baseline and 2-year follow-up, *p* p-value of paired t-test, *SAS* Stand Up and Sit Down, *SLW* Short Line Walk, *SCSW* Short Comfortable Speed Walk, *SMSW* Short Maximum Speed Walk, *SIP* Stepping in Place, *POCO* Postural Control, *RR* Romberg Ratio, *ce* closed eyes, *oe* open eyes, *n.u.* unitless

## Discussion

In this longitudinal observation study of 100 pwMS with predominantly low to moderate disability levels, we explored patterns of change and sensitivity of 26 DMO of gait and balance against EDSS progression events and, in a novel approach, a PRO anchor.

In accordance with prior evidence [4, 39], the number of EDSS progression events was expectedly low with 22 (23.16%) and 11 (15.71%) MSWS-12 progression events. Of note, both subgroups showed distinct motor characteristics (Fig. 3), likely reflecting clinical differences (see Table 1): MSWS-12 progression events occurred from higher baseline disability, while nine out of 22 subjects transitioned from EDSS baseline scores of zero. This inconsistency points to conceptual differences between anchors in which lower EDSS ranges are dominated by non-ambulation symptoms, whereas MSWS-12 focuses on gait, a relevant symptom in higher EDSS.

The primary analysis (LMM) yielded two major findings. First, at the group level, our study population showed expectedly small changes in DMO over the 2-year observation period, although with high variance. These findings align with the low proportion of progression events observed by clinical outcomes. Still, six of the 26 motor outcomes showed changes over time that were robust by magnitude. The direction of change was clinically plausible and suggests deterioration in postural control, step symmetry in walking and possibly limb strength. Interestingly, these outcomes differed from those that primarily distinguish pwMS from healthy controls in the normative data set (Fig. 1).

Second, our analyses identified step width (SCSW) and 3D sway speed (POCO) as potential early indicators of progression. Subjects with EDSS progression, a subgroup featuring only minimal baseline disability, showed approximately three times greater changes than those without. This remarkable finding suggests that these motor outcomes, both related to functions of postural control, may serve as sensitive indicators of early disease-related changes in MS. Notably, maximum gait speed did not change over time or in relation to a progression event, but differed significantly between those with and without progression. This suggests its potential as an indicator of MS-related disability, particularly in mildly disabled cohorts, rather than a dynamic marker of change over time. The interaction effect for asymmetry while stepping in place was unexpected in direction, as were the only two interaction effects of time and progression event classified by MSWS-12. Post-hoc model diagnostics revealed slight violations of homoscedasticity for these parameters, which are in line with previously reported skewness in their distributions [18]. Although model fit was deemed acceptable, this may have affected estimates' robustness and should be considered in interpretation.

Our findings of differing patterns between DMO with main time effects and those with interaction effects align with previous research. One study found a deterioration in gait and balance measures over a 12-month period in patients with EDSS scores $\le 3$ that was not convergent with concurrent changes in EDSS [7]. Others demonstrated changes in key spatio-temporal gait parameters over time, which were not correlated with changes in EDSS but with changes in MSWS-12 [10]. Thus, our results strengthen the concept of DMO as sensitive markers of early disease-related changes in motor performance that are not (yet) detectable in EDSS. This also implies that EDSS may not be the most appropriate anchor to define minimal clinically important difference (MCID) but rather changes of robust clinical relevance.

To our knowledge, we are the first to report MDC values for DMO. The MDC serves as a metric of method’s reliability and thus an important reference to interpret DMO changes with potential clinical relevance according to clinical anchors. Of note, changes below this threshold may theoretically be clinically relevant but not reliably detectable with the motion capture system used in this study [40]. For EDSS, two of the three DMOs that showed significant changes in relation to a progression event also surpassed the MDC, reflecting reliable change beyond measurement error, further supporting the LMM analysis. Usually, the difference between group means for those with versus without progression event is taken as a useful estimate of the clinical relevance of change; for example, changes of 0.7 SD in step width or > 1SD for 3D sway speed (oe) could be considered clinically important anchored by EDSS. While this study defines DMOs related to balance functions as concepts of interest for the assessment of disability progression in MS, future studies in heterogeneous cohorts and various anchors, such as the global impression of change, will help to corroborate MCID values for the clinical use of a disease-specific set of DMOs. Of note, the use of MDC is limited for results from distribution-based analysis performed in the same sample and by the annual measurement intervals but still provides a valuable reference for future study in different samples.

### Contextualization

Our study goes beyond previous work by validating DMO against established clinical progression criteria and modeling their responsiveness to change over time. Further study needs to confirm the predictive value of such markers for clinically relevant progression events and investigate determinants more widely. For example, own results showed effects of fatigue on motor outcomes of balance [41].

So far, despite regulatory frameworks in place, there appears to be no methodological consensus regarding the validation of DMO. From our journey in evaluating PASS-MS, their implementation requires strict protocol consistency, adherence, and standardized data processing including post-hoc quality control. The exclusion rate of 8.3% supports the reliability of our measurement protocol; however, this is from mono-center application only. Current endeavors in this respect include smartphone-based protocols [8], which have been extended to standardized remote assessments currently under longitudinal investigation. For the RGB-D protocol used herein, the HOPE study [42] investigates self-administered motor assessment protocols similar to our study, while others focus on developing and evaluating wearable sensors for mobility assessments across various conditions, including MS [43, 44].

### Limitations and outlook

The sample size is large compared to other DMO validation studies but remains a limiting factor for modeling effects. The small number of progression events further limited statistical power, specifically for the PRO anchor. The cohort’s characteristics may reduce generalizability to more advanced MS populations. Sufficiently long observation periods or larger samples are needed to achieve more robust results. A notable proportion of EDSS progression events represents conversions from EDSS 0 to 1 or higher, which may reflect subtle changes rather than functional worsening. Given that Kinect v2 was discontinued in 2017, the direct applicability of our results is limited. While our analysis algorithms were designed to be sensor-agnostic and the *z*-score-based approach allows for direct comparability between devices, reproducibility across hardware generations and clinical settings remains to be demonstrated.

Our study provides initial estimations of DMO responsiveness by focusing on clinical progression irrespective of relapse status. Further research should more comprehensively investigate the differential utility of DMO in capturing PIRA- and RAW-related changes, additionally integrating disease activity markers, such as MRI or serum biomarkers. Moreover, multiple clinical and patient-reported anchors should be applied to derive robust estimates of meaningful change.

## Conclusions

We demonstrated systematic changes in DMO in relation to a clinical progression event. Additionally, subtle changes that may not be captured by conventional clinical measures such as the EDSS became apparent. This is of significant clinical relevance, as a stable EDSS score does not necessarily indicate the absence of underlying motor decline. Though further investigation is needed to confirm our findings and to more reliably define meaningfulness against patient-reported changes, our findings emphasize the potential of DMO as sensitive progression markers for disease monitoring in MS.

### Supplementary material

Supplemental material for this article is available online. Supplementary tables and figures referenced in the manuscript are provided in a separate file and are based on data already presented in the main text. The datasets generated during and/or analyzed during the current study are not publicly available due to restricted consent and data protection regulations in the country of the study site.

## Supplementary Information

Below is the link to the electronic supplementary material.

Supplementary file 1 (PDF 1458 KB)

## Data Availability

The datasets generated during and/or analyzed during the current study are not publicly available due to restricted consent and data protection regulations in the country of the study site.
