# Supplementary material for: Anchor-based evaluation of digital motor biomarkers from a 2-year observation in 100 patients with multiple sclerosis
Source: J Neurol. 2025 Dec 4;273(1):4. doi: 10.1007/s00415-025-13541-y (PMC12678454; doi:10.1007/s00415-025-13541-y)

Anchor-based evaluation of digital motor biomarkers from a two-year observation in 100 patients with multiple sclerosis

Journal of Neurology

Supplementary material

Corresponding author:
M.Sc.Psych. Carla A. Ellinghaus
Experimental and Clinical Research Center, Charité - Universitätsmedizin Berlin
E-mail address: carla.ellinghaus@charite.de

Supplementation of missing follow-up visits

Due to institutional restrictions on patient visits in context of research projects during the COVID-19 pandemic, no study visits could be conducted between March 2020 and July 2020, from December 18, 2020, to March 1, 2021, and from January 24, 2022, to February 14, 2022. During the remaining periods, visits were conducted to a limited extent. In addition, there were increased and unforeseeable cancellations of visits by participants due to the pandemic. We were able to follow up on 52 participants after the first year and 59 participants after the second year.

As data on start and end point are indispensable for our investigation and statistical analysis, missing follow-up data were supplemented by using one or two visits preceding baseline, or one or two visits following the last available timepoint. Supplementary data from the same individuals were obtained from aligned observational studies applying an identical protocol (see Ethics). In total, 64 visits were supplemented, corresponding to 43 cases. Of the 100 original baseline visits, 38 were reassigned to serve as follow-up visits (6 as 1-year follow-up and 32 as 2-year follow-up) and supplemented by earlier assessments. In five cases, 2-year follow-up was not available. Here, the first follow-up was used as baseline, the second as 1-year follow-up and a later visit was added for two-year follow up. After supplementation, complete data were available for 100 baseline visits, 63 1-year follow-up visits and 96 two-year follow-up visits (Supplementary Fig. S1).

Supplementary Fig. S1 Flowchart illustrating patient follow-up and data supplementation

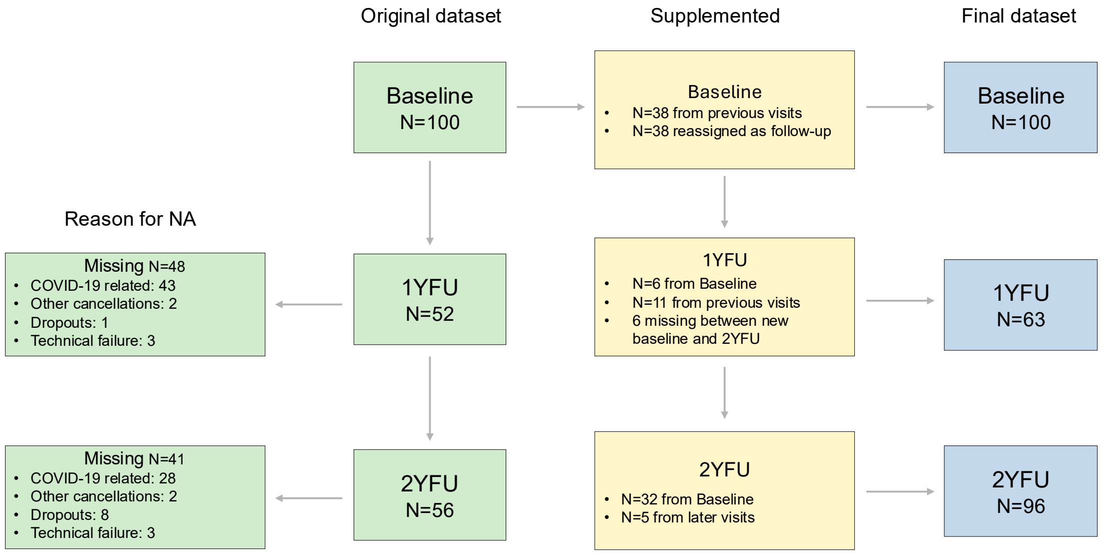

1YFU 1-year follow-up, *2YFU* 2-year follow-up, *NA* not available

Supplementary Table S1 Number of excluded recordings and cases per test due to quality concerns over the entire observation period

Test	Recordings<br>N	Excluded recordings<br>N (%)	Excluded subjects<br>N
**SAS**	745	34 (4.6)	19
**SLW**	690	44 (6.4)	27
**SCSW**	742	79 (10.7)	40
**SMSW**	730	48 (6.6)	25
**SIP**	245	39 (15.9)	39
**POCO**	244	38 (15.6)	38

SAS Stand Up and Sit Down, *SLW* Short Line Walk, *SCSW* Short Comfortable Speed Walk, *SMSW* Short Maximum Speed Walk

Supplementary Fig. S2 Correlation matrix of the original z-standardized parameter set

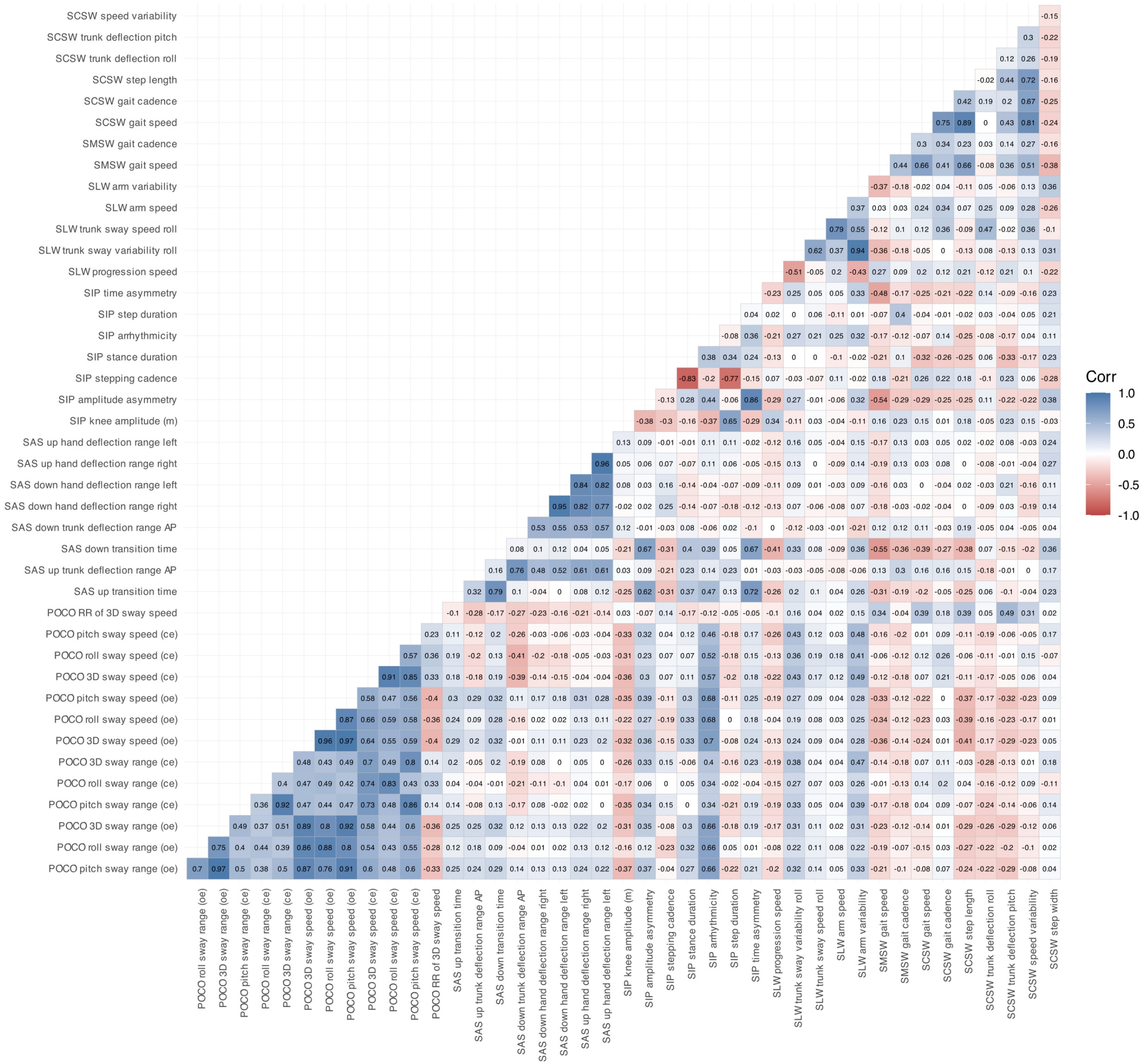

Supplement: Supplementary file 1 — (PDF 1458 KB) [file 415_2025_13541_MOESM1_ESM.pdf]
